# Analysis of *Zobellella denitrificans* ZD1 draft genome: Genes and gene clusters responsible for high polyhydroxybutyrate (PHB) production from glycerol under saline conditions and its CRISPR-Cas system

**DOI:** 10.1371/journal.pone.0222143

**Published:** 2019-09-12

**Authors:** Yu-Wei Wu, Shih-Hung Yang, Myung Hwangbo, Kung-Hui Chu

**Affiliations:** 1 Graduate Institute of Biomedical Informatics, College of Medical Science and Technology, Taipei Medical University, Taipei, Taiwan; 2 Clinical Big Data Research Center, Taipei Medical University Hospital, Taipei, Taiwan; 3 Zachry Department of Civil and Environmental Engineering, Texas A&M University, College Station, TX, United States of America; Academia Sinica, TAIWAN

## Abstract

Polyhydroxybutyrate (PHB) is biodegradable and renewable and thus considered as a promising alternative to petroleum-based plastics. However, PHB production is costly due to expensive carbon sources for culturing PHB-accumulating microorganisms under sterile conditions. We discovered a hyper PHB-accumulating denitrifying bacterium, *Zobellella denitrificans* ZD1 (referred as strain ZD1 hereafter) capable of using non-sterile crude glycerol (a waste from biodiesel production) and nitrate to produce high PHB yield under saline conditions. Nevertheless, the underlying genetic mechanisms of PHB production in strain ZD1 have not been elucidated. In this study, we discovered a complete pathway of glycerol conversion to PHB, a novel PHB synthesis gene cluster, a salt-tolerant gene cluster, denitrifying genes, and an assimilatory nitrate reduction gene cluster in the ZD1 genome. Interestingly, the novel PHB synthesis gene cluster was found to be conserved among marine Gammaproteobacteria. Higher levels of PHB accumulation were linked to higher expression levels of the PHB synthesis gene cluster in ZD1 grown with glycerol and nitrate under saline conditions. Additionally, a clustered regularly interspaced short palindromic repeat (CRISPR)-Cas type-I-E antiviral system was found in the ZD1 genome along with a long spacer list, in which most of the spacers belong to either double-stranded DNA viruses or unknown phages. The results of the genome analysis revealed strain ZD1 used the novel PHB gene cluster to produce PHB from non-sterile crude glycerol under saline conditions.

## Introduction

Polyhydroxybutyrate (PHB) is a promising alternative to petroleum-based plastics because PHB is biodegradable [1, 2] and can be produced from renewable materials by a number of microorganisms [3–5]. However, the price of PHB is less competitive to the petroleum-based plastics. Using sterile expensive substrates to cultivate PHB-producing microbes is a major factor contributing to the overall high PHB production cost [6]. One approach to overcome this challenge is to identify organisms that are capable of producing PHB from inexpensive waste organics, followed by a better understanding of their PHB biosynthesis pathways which can be used to optimize PHB production from organic wastes.

Our previous study reported that *Zobellella denitrificans* ZD1 (referred as strain ZD1 hereafter) can accumulate high contents of PHB from both sterile and non-sterile synthetic crude glycerol (containing fatty acids and salts) [7]. Crude glycerol, a biodiesel production byproduct that is expected to be available in a large quantity, is an inexpensive (0.11 US$/kg) and promising sustainable carbon source for PHB production. Strain ZD1 is a heterotrophic, gram-negative rod bacterium [8] isolated from a mangrove ecosystem. Along with another isolate *Z*. *taiwanesis* ZT1, strain ZD1 and *Z*. *taiwanesis* ZT1 are the first two members of the *Zobellella* genus belonging to the Gammaproteobacteria class. They are capable of using nitrite and/or nitrate as electron acceptors in their respiratory and fermentative metabolism [8].

Herein, built on our recently assembled draft genome of strain ZD1 [7], we reported genome analytical results of the strain ZD1 with a focus on identification of genes and gene clusters associated with glycerol utilization, PHB production and salt tolerance in the ZD1 genome. We reported that strain ZD1 has a novel PHB-synthesis gene cluster which is only partially similar to the gene cluster identified previously [9, 10]. We also conducted a comprehensive search and evaluation of this gene cluster among existing bacterial genomes. We also identified a three-gene cluster (*ectA*, *ectB*, and *ectC*) responsible for the synthesis of ectoine (1,4,5,6-tetrahydro-2-methyl-4-pyrimidinecarboxylic acid) in the ZD1 genome. Ectoine is a common osmolyte in many halophilic eubacteria. Introduction of this gene cluster into another non-salt-tolerant species also increases its ability to withstand a salty environment [11], supporting the hypothesis that ZD1 can withstand high salinity environment in liquid medium, as previously described [8].

Our recent work has observed significant effects of nitrogen sources on PHB production by strain ZD1 [12]; therefore the relationship between PHB gene expression and nitrogen sources were scrutinized in more details in this study. Gene clusters that are relative to various metabolic pathways such as denitrification and nitrite reduction were also identified. A clustered regularly interspaced short palindromic repeat (CRISPR)-Cas system that is structurally very similar to that of *Escherichia coli* was identified in the ZD1 genome and compared among closely related species.

## Materials and methods

### Bacterial strain and chemicals

*Zobellella denitrificans* ZD1 (JCM 13380) were purchased from Riken BRC Microbe Division, Japan Collection of Microorganisms (JCM). FastRNA^™^ Pro Blue Kit and Isopropyl-β-D-1-thiogalactopyranoside (IPTG) was purchased from MP Biomedicals (Santa Ana, CA, USA). Imperial^TM^ protein stain was purchased from Fisher Scientific (Waltham, MA, USA). OneStep Ahead RT-PCR Kit was obtained from Qiagen (Hilden, Germany). Power SYBR Green PCR Master Mix was purchased from Applied Biosystem (Waltham, MA, USA).

### Culture conditions, PHB measurement and RNA extraction

Strain ZD1 was grown aerobically in ammonium ((NH_4_)_2_HPO_4_) and nitrate (KNO_3_) modified mineral salts medium, respectively, supplied with 20 g/L glycerol and 0% or 3% NaCl (w/v) to above an optical density 600 nm (OD_600_) of 1.0 (i.e., in stationary growth phase) before harvested for PHB genes and salt tolerance gene analysis. The modified mineral salts medium contained K_2_HPO_4_ (5 g/L), Na_2_SO_4_ (0.5 g/L), MgSO_4_·7H_2_O (0.4 g/L), and 0.1% (v/v) trace mineral solution. The trace mineral solution contained FeSO_4·_7H_2_O (2.78 g/L), MnCl_2_·4H_2_O (1.98 g/L), CoSO_4_·7H_2_0 (2.81 g/L), CaCl_2_·2H_2_O (1.47 g/L), CuCl_2_·2H_2_O (0.17 g/L), and ZnSO_4_·7H_2_O (0.29 g/L). Total RNA was extracted using FastRNA^™^ Pro Blue Kit according to the manufacturer’s instruction. PHB concentration in dry cell weight (DCW) was determined using a spectrophotometric assay via conversion of PHB to crotonic acid as described previously [13–15].

### Quantification of PHB synthesis genes (*phaA*, *phaB*, *phaC* and *PFP*) from cDNA

The cDNA was synthesized using OneStep Ahead RT-PCR Kit according to the manufacturer’s instruction. Briefly, 1μg of total RNA was added to a 25-μL reaction mix containing Omniscript^®^ and Sensiscript^®^ reverse transcriptases, DNA polymerases, 0.5 μM primers (listed in S1 Table), 2.5 mM MgCl_2_ in RNAase free water. The cDNA was obtained by the following cycling condition: 10 min of reverse transcription at 50 ^o^C, 5 min of DNA polymerase activation at 95 ^o^C, followed by 40 cycles of three-step amplification (95 ^o^C for 20 sec, 54 ^o^C for 20 sec and 72 ^o^C for 20 sec, and a final extension at 72 ^o^C for 2 min). The cDNA was stored at -80 ^o^C before used. The cDNA was diluted 200 times before real-time quantitative PCR for PHB synthesis genes and a housekeeping gene (16S rRNA) using an IQ5 multicolor real-time PCR detection system (Bio-Rad, Hercules, CA, USA). Primer sets specific to the PHB synthesis genes and housekeeping gene were listed in S1 Table. All of the primers were synthesized by Integrated DNA Technologies (Coralville, IA, USA). Briefly, the reaction mix contained 1 μL of diluted cDNA, 10 μL of PowerSYBR Green PCR Master Mix, 0.5 μM of each of the primer sets and nuclease-free water to 20 μL. The cycling condition was conducted in 1 cycle of denaturation at 95°C for 10 min, followed by 35 cycles of three-step amplification (i.e., 95°C for 30 sec, followed by 54°C (PHB synthesis genes) or 55°C (housekeeping gene) for 45 sec, and then 72°C for 45 sec). The fluorescence intensity was measured during each three-step cycle. The Ct value, defined as the PCR cycle number that resulted in exceeding an arbitrarily signal threshold, was used as the endpoint of the real-time PCR quantification. Gene expression was calculated by Double delta Ct method as described in previous study [16]. The fold change of ZD1 in nitrate treatment was normalized to that observed in ammonium treatment.

### Sodium dodecyl sulfate polyacrylamide gel electrophoresis (SDS-PAGE) Analysis

SDS-PAGE analysis was performed to examine the expression of genes (*ectA*, *ectB*, and *ectC*) responsible for ectoine synthesis by strain ZD1 and compared to over-expression of each gene in the plasmid by *E coli*. Over-expression of these genes were induced by adding 0.2 mM IPTG to *E*. *coli* BL21 (DE3) that contains a plasmid of each of three genes constructed previously [12]. Briefly, *ectA*, *ectB*, and *ectC* in strain ZD1 were cloned into three pET11a vectors, using traditional cloning with T4 DNA ligase (New England Biolabs, Ipswich, MA, USA). The constructed plasmids were transformed into *E*. *coli* competent cells (NEB 5-alpha), followed by screening on LB agar plates containing 100 mg/L ampicillin. The plasmids of the colonies on the plates were then extracted by QIAprep Spin Miniprep Kit (QIAGEN, Hilden, Germany), followed by sequence confirmation. The correct plasmids were then transformed into *E*. *coli* BL21 for protein expression. The *E*. *coli* BL21 and ZD1 cultures were concentrated to OD_600_ around 16.7 in sodium phosphate buffer (MP Biomedicals, Santa Ana, CA, USA) and lysed by sonication. Sonication was conducted in 6 rounds of 20 sec sonication (50% amplification) and 20 sec on ice intervals. A 15 μL supernatant of lysed cultures was mixed with 15 μL of 2X SDS buffer in a 1.5-mL microcentrifuge tube and then boiled for 10 min. SDS-PAGE was conducted on Novex^™^ wedge well 14% tris-glycine gel (Invitrogen, Carlsbad, CA, USA) in XCellSureLock^™^ Mini-Cell equipment (Novel Experimental Technology, San Diego, CA, USA). 1X Tris-Glycine (MP Biomedicals, Santa Ana, CA, USA) running buffer and 10 μL of the Mark12 Unstained Standard (Invitrogen, Carlsbad, CA, USA) were used. The electrophoresis was run at 90 V for 2.5 hrs. The gel was stained with Imperial^TM^ protein stain for 1 hr, and then destained for 2 hrs with deionized water.

### *Zobellella denitrificans* ZD1 Genome Analysis

The draft genome of strain ZD1 [7] was used for a phylogenetic analysis. A phylogenetic tree was constructed using ezTree [17] (https://github.com/yuwwu/ezTree), which was capable of mining single copy marker genes and generating phylogenetic trees from the concatenated alignment of the marker genes, and was visualized using MEGA5 [18]. Genes were predicted using the NCBI Prokaryotic Genome Annotation Pipeline (PGAP) [19] and used in a downstream functional analysis. Sequences for building the phylogenetic tree of the *norB* genes that code nitric oxide reductase [20] were downloaded from the NCBI database (https://www.ncbi.nlm.nih.gov/) according to their accession numbers and aligned using MUSCLE [21]. The phylogenetic tree was then built using FastTree [22] with JTT model.

To probe the existence of gene clusters among the Bacteria kingdom, 36,682 bacterial genomes were downloaded from the NCBI ftp site (ftp://ftp.ncbi.nlm.nih.gov/genomes/) in April 2018. Only the best-assembled strain (i.e., with the highest N50 length) of each species was retained for analysis. A total of 8,482 genomes were kept in this process. Genes were predicted from the selected genomes using Prodigal [23], and the presence of the PHB gene cluster was checked using BLASTP [24]. The phylogenetic tree of PHB gene clusters was made by separately aligning individual genes using MUSCLE [21], concatenating the alignments, and then building the tree using FastTree [22] with JTT model. The tree was then plotted and colored using the Evolview web service [25]. The conserved functional domains of phasin proteins were obtained by searching against NCBI Conserved Domain Database (CDD) [26] using the NCBI CD-Search service [27]. The phylogenetic tree of phasin proteins was constructed by first aligning genes using MUSCLE, followed by building the tree using FastTree with the JTT model, and then visualized using MEGA5 [18].

Clustered regularly interspaced short palindromic repeat (CRISPR)-Cas systems were checked using the CRISPRone web service [28]. The taxonomic distribution of CRISPR spacers was obtained as described by Shmakove et al [29]. Briefly, a total of 154,924 viral sequences was downloaded from the NCBI genome website (https://www.ncbi.nlm.nih.gov/genome/viruses/) in June 2018. The CRISPR spacers were then searched against the downloaded viral sequences using BLASTN with the following parameters: “-dust no -word_size 8 -max_target_seqs 1 -outfmt 6.” Only hits with at least 95% identity were parsed to identify the taxonomic profiles of the most closely related phages.

## Results

**Fig 1:**
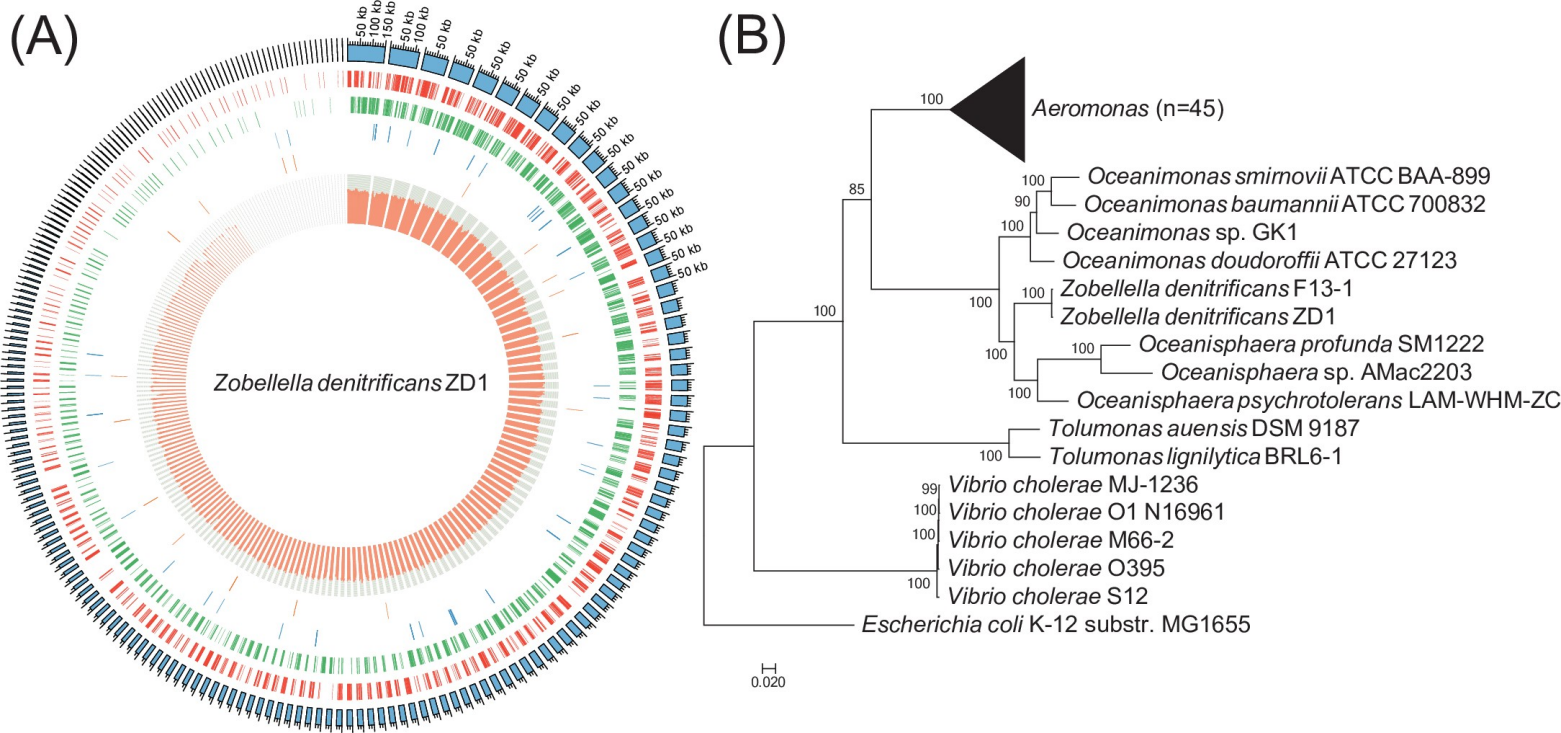
Genome map and phylogenetic tree for *Zobellella denitrificans* ZD1 strain. (A) Six rings, from outer to inner, represent (1) assembled scaffolds, (2) genes in the forward strand, (3) genes in the reverse-complement strand, (4) transfer RNA (tRNA) genes, (5) ribosomal RNA (rRNA) genes, and (6) the GC content. (B) Phylogenetic tree constructed from 287 single copy marker genes.

Assembly of the strain ZD1 draft genome yielded 217 scaffolds [7] (Fig 1A). The genome size of ZD1 was 4,501,699 base pairs (bp), and the overall GC content was 63.78%. The length of the longest scaffold was 150,144 bp, and the N50 length was 33,403 bp. The phylogenetic tree, as shown in Fig 1B, was constructed using 287 single copy marker genes (listed in S2 Table) identified by the ezTree pipeline. Our results suggest that strain ZD1 is most closely related to species of *Oceanisphaera* and *Oceanimonas*, consistent with the 16S ribosomal RNA tree reported previously [8] (The relationship between *Z*. *denitrifica*ns and *Z*. *taiwanensis*, the first two members of the *Zobellella* genus, can be seen in S1 Fig). The bootstrap confidence levels of most of the branches on this tree exceeded 0.99, indicating that the constructed phylogenetic tree was trustworthy.

### Glycerol utilization and PHB production

**Fig 2:**
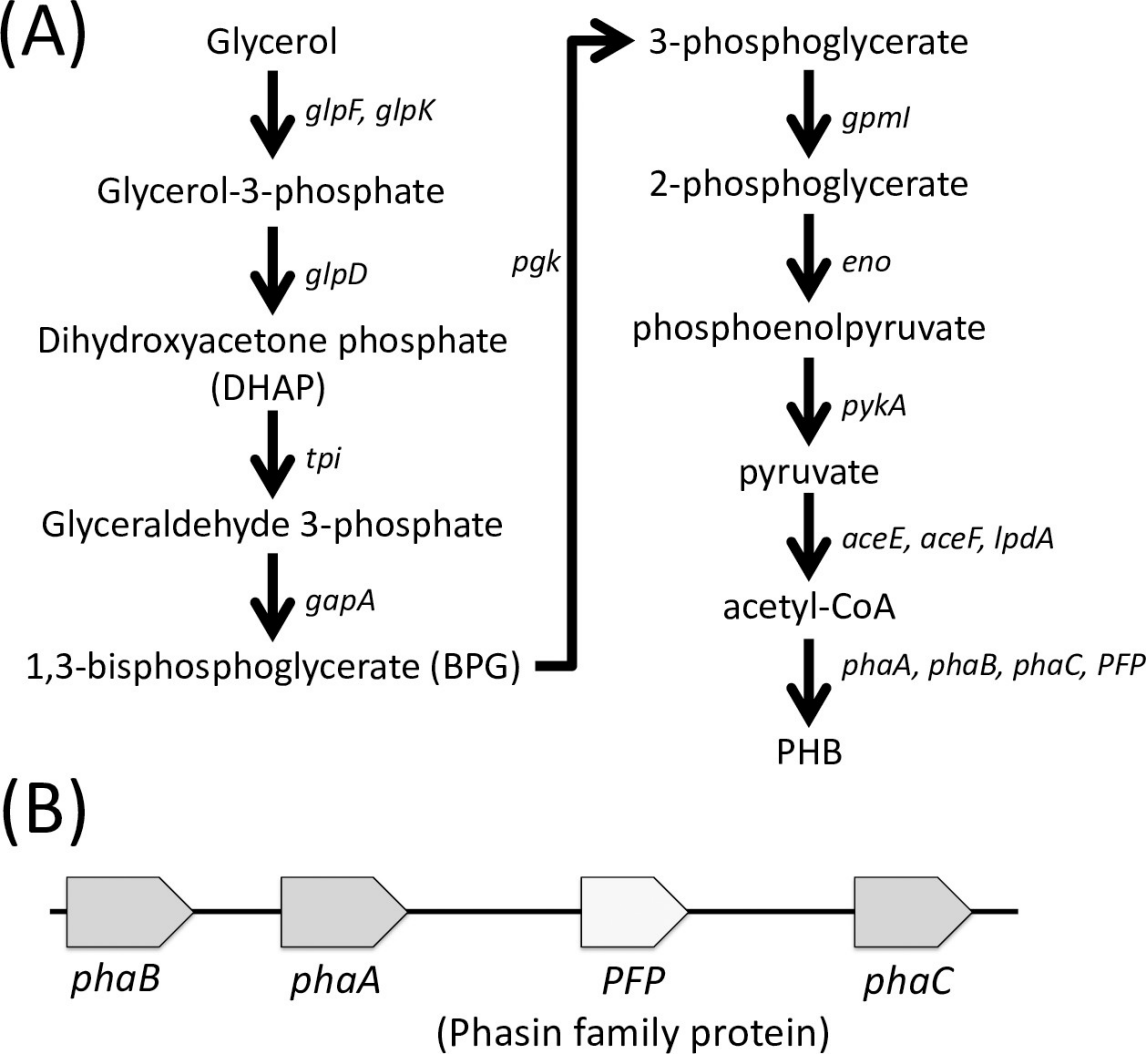
**(A) A proposed pathway to convert glycerol to polyhydroxybutyrate (PHB). (B) The PHB synthesis gene cluster identified in the genome of *Zobellella denitrificans* ZD1.** The pathway figure was adapted from Martinez-Gomez et al. [34].

A complete pathway for converting glycerol to PHB was identified in the ZD1 genome (Fig 2). As shown in Fig 2A, series of reactions and enzymes are involved in the uptake and conversion of glycerol to PHB. Briefly, *glpF* encoding glycerol facilitator is responsible for uptake of glycerol into cells and *glpK* encoding glycerol kinase converts glycerol to glycerol-3-phosphate. Glycerol-3-phosphate is then converted to dihydroxyactonephosphate (DHAP) and eventually to pyruvate. Pyruvate is then oxidized to acetyl-CoA, which is converted into PHB. A PHB synthesis gene cluster, consisting of four genes: *phaB*, *phaA*, *PFP* (a phasin-family protein gene), and *phaC*, was shown in Fig 2B. The three genes (*phaA*,*-B*, and *-C*) encoding enzymes that are known to convert acetyl-CoA into PHB, in which PhaA served as the first enzyme to catalyze acetyl-CoA to acetoacetyl-CoA, PhaB transforms acetoacetyl-CoA to (R)-3-hydroxybutyryl-CoA, and PhaC catalyzes the polymerization and formation of PHB granule [30]. The *PFP* may also play a role in the formation of the PHB granule [31]. Other phasin-encoding genes, including *phaP* and *phaF* [32], along with a *phaR* gene (a phasin transcription factor known to bind to the *phaP* promoter region and block *phaP* expression [33]) were also found in the genome.

**Fig 3:**
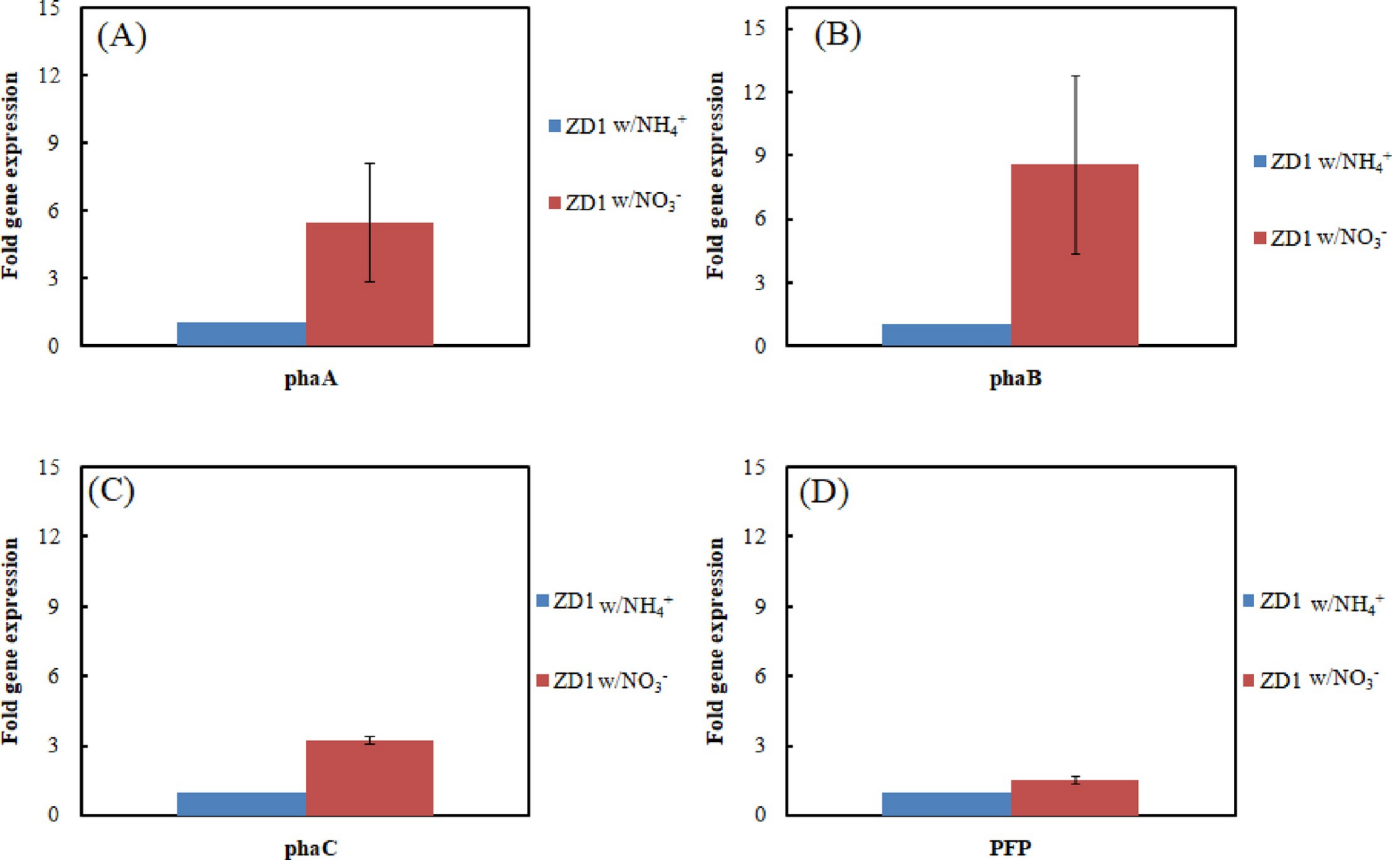
Effects of ammonium (NH_4_^+^) and nitrate (NO_3_^-^) on the expression of PHB synthesis genes. (A) *phaA* (B) *phaB* (C) *phaC* and (D) *PFP* in *Zobellella denitrificans* ZD1.

We further examined the relationship between PHB accumulation and expression of PHB synthesis genes in strain ZD1 grown with two different nitrogen sources, ammonia and nitrate. Strain ZD1 accumulated 9.5 g/L and 16.3 g/L of PHB when aerobically grown with ammonium and nitrate, respectively. Higher mRNA levels encoding *phaA* (2.5 ~ 8.1 fold increase), *phaB* (4.4 ~12.8 fold increase), and *phaC* (3.1~ 3.4 fold increase) were observed in nitrate-grown cultures compared to those grown with ammonium (Fig 3). The *PFP* expression level was also slightly increased in the nitrate-grown cultures (increased 0.8–2.2 fold). These results confirmed that the PHB synthesis gene cluster encoding enzymes were responsible for PHB accumulation in strain ZD1. The high expression levels of *phaA* and *phaB* in the nitrate-grown cultures were unclear; however, they might be explained by the differences in nitrogen metabolism of the strain.

### Distribution of the PHB gene cluster among bacteria

**Fig 4:**
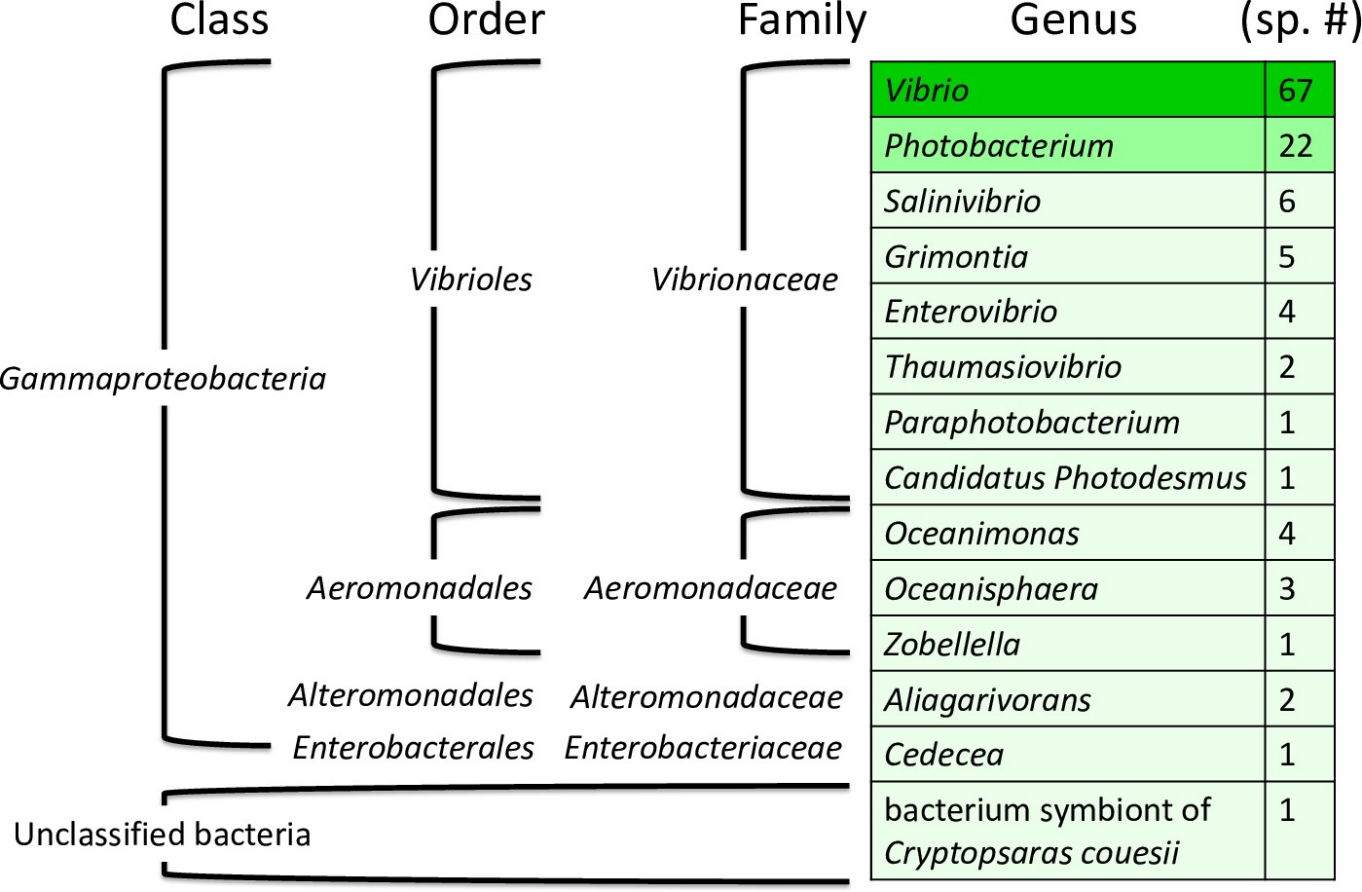
Distribution of the bacterial species harboring a polyhydroxybutyrate (PHB) synthesis gene cluster (*phaB*-*phaA*-*PFP*-*phaC*) similar to that of the *Zobellella denitrificans* ZD1. Genus name and the family, order, and class ranks are indicated accordingly. Sp # refers to the number of bacterial species containing the PHB synthesis gene cluster. Only one strain per species was used in the calculation. See "Materials and Methods" for details.

The PHB synthesis gene cluster consists of four genes: *phaB*, *phaA*, *PFP* (a gene encoding a phasin-family protein), and *phaC*. The order of the PHB synthesis gene cluster (i.e., *phaB*, -*A*, and -*C* genes) is similar to that of *Azotobacter* sp. FA8 [35]. However, to the best of our knowledge, the *PFP* between *phaC* and *phaA* in this gene cluster has not been previously documented. After searching for this four-gene-cluster in genomic sequences in the NCBI database, 117 species containing this gene cluster were identified (Fig 4). The majority of these species were Gammaproteobacteria, belonging to the *Vibrionaceae* family, in which the *Vibrio* genus accounts for 55.6% of species, followed by *Photobacterium* (18.8%). Only eight species (four *Oceanimonas*, three *Oceanisphaera*, and the *Z*. *denitrificans* species) were in the *Aeromonadaceae* family to which strain ZD1 belongs to. Almost all of these species were marine microorganisms and thus halophilic, suggesting that this gene cluster may specifically belong to ocean-dwelling bacterial organisms.

**Fig 5:**
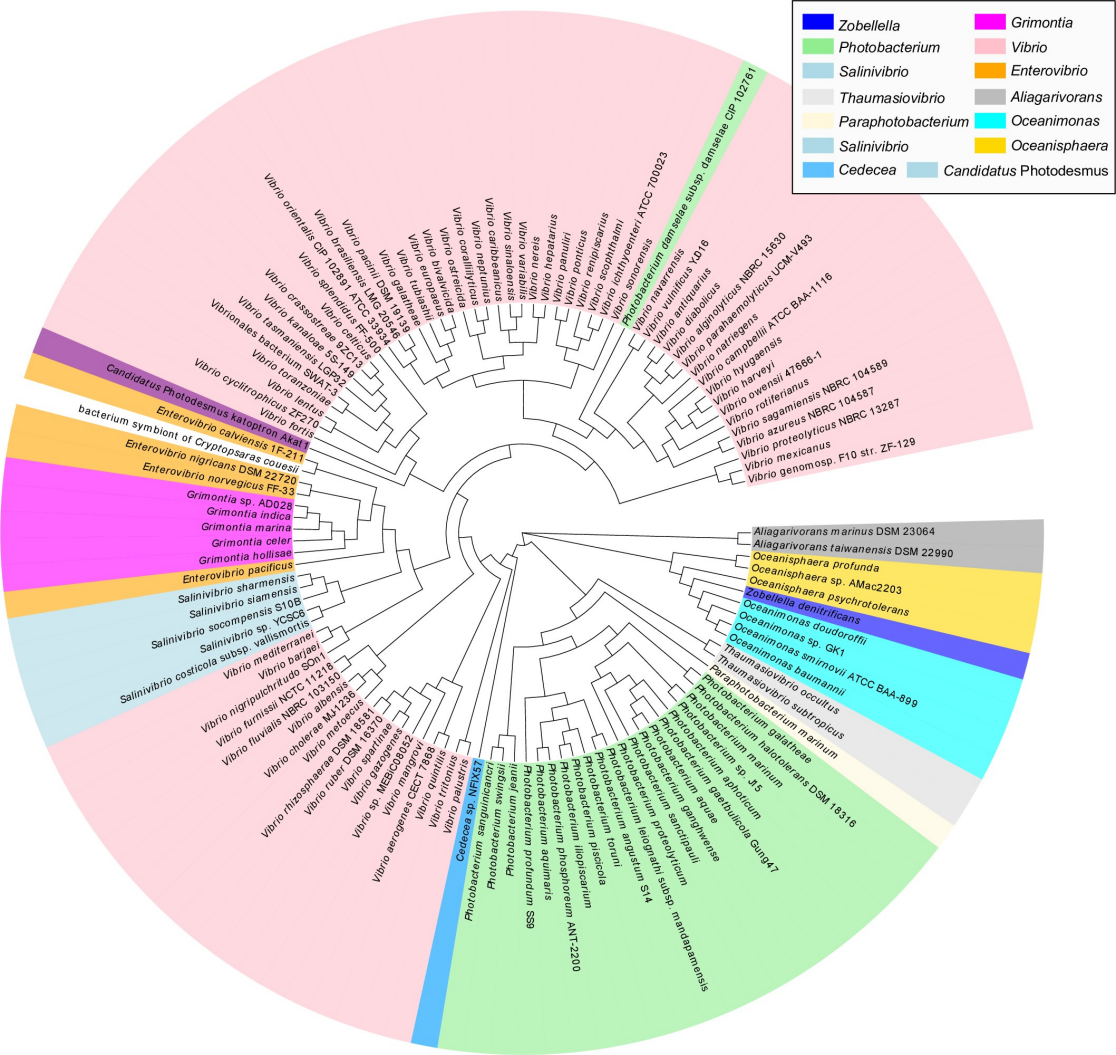
Concatenated protein tree built from four genes of the PHB gene cluster. The same color indicates the same genus rank. *Zobellella denitrificans* is indicated by blue color on the right side.

To explore evolutionary relationships among these 117 species, a phylogenetic tree was constructed from the concatenated amino acid sequences of the four genes in the PHB synthesis gene cluster (Fig 5). The *Aeromonadales* tree topology (including *Oceanimonas*, *Oceanisphaera*, and *Zobellella*) along with the order *Alteromonadales* generally agrees with the phylogenetic tree based on 16S rRNA gene (Fig 1B). However, several horizontal gene transfers seem to have occurred in the order *Vibrioles*. The most visible one might be a transfer event from *Vibrio* to the common ancestor of *Salinivibrio*, *Grimontia*, and *Enterovibrio*. A bacterial symbiont of the triplewart seadevil (*Cryptopsarascouesii*) and a *Photobacterium* species (*Photobacterium damselae* subsp. *damselae* CIP 102761) may have acquired the cluster from *Enterovibrio* and *Vibrio*, respectively. The observation suggests that this PHB synthesis gene cluster may have occasionally been horizontally transferred among different marine microorganisms.

The *PFP* in ZD1 was also investigated. A *phaP* gene was identified in the ZD1 genome and the *phaP* is similar to those encoding phasin proteins. The PHB synthesis gene cluster encoding proteins were assigned to the conserved protein domain family phasin_2_superfamily (cl11491), which is designated as “associated with polyhydroxyalkanoate (PHA) inclusions, commonly consisting of polyhydroxybutyrate (PHB) (https://www.ncbi.nlm.nih.gov/Structure/cdd/cddsrv.cgi?uid=325059)” (S2A Fig). The phylogenetic tree demonstrates that in their evolutionary history, the *PFP* identified from the ZD1 PHB gene cluster may have diverged from the *phaP* clade (S2B Fig).

### Salt tolerance

**Fig 6:**
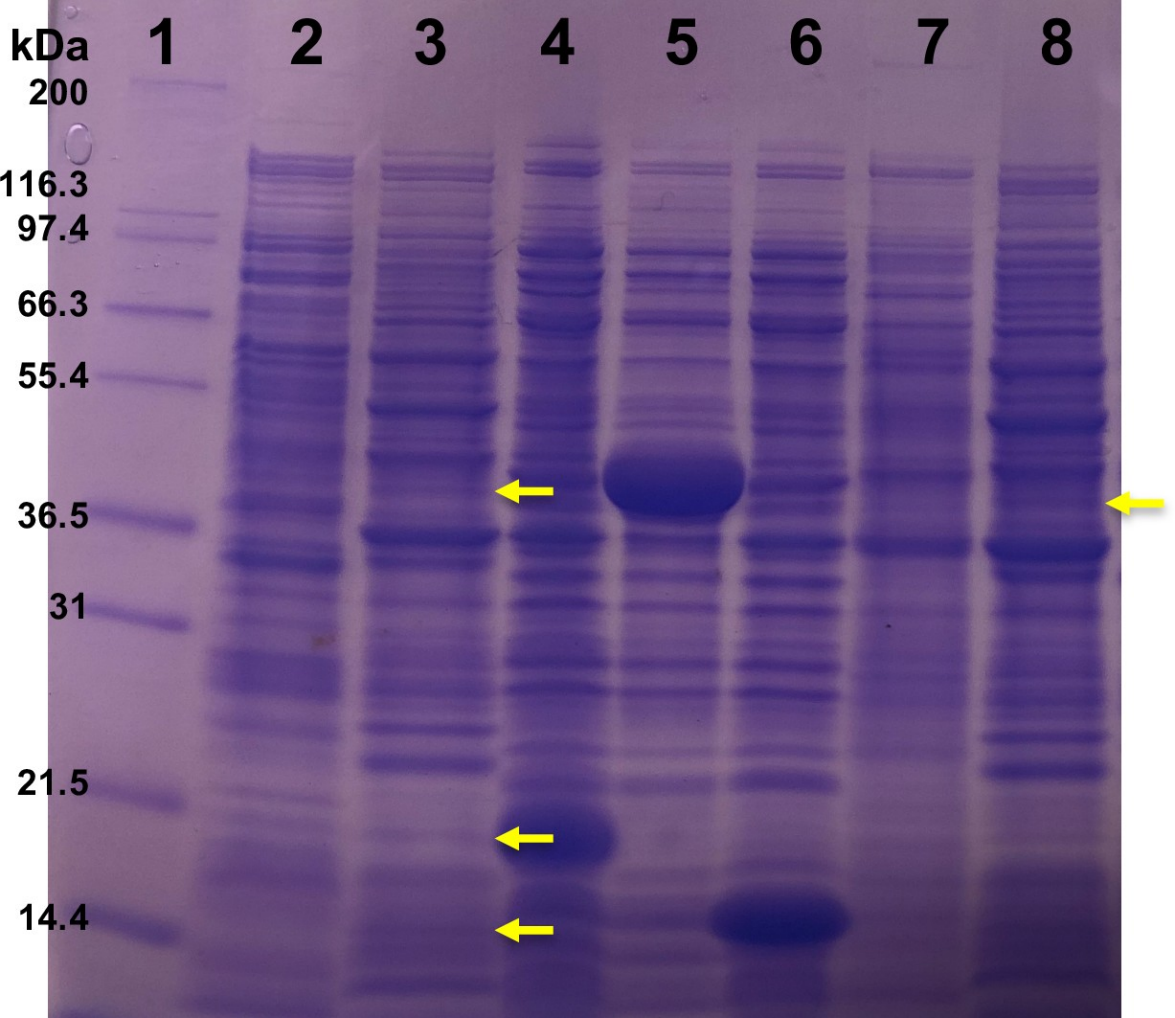
SDS-PAGE analysis of three proteins (EctA, EctB, and EctC) produced in *Zobellella denitrificans* ZD1 and in *E*. *coli* BL21. **1**: Mark12 Unstained Standard; **2**: supernatant of lysed cells grown with ammonia and 0% NaCl; **3**: supernatant of lysed cells grown with ammonia and 3% NaCl; **4**: EctA produced by *E*. *coli* after 0.2 mM IPTG induction; **5**: EctB produced by *E*. *coli* after 0.2 mM IPTG induction; **6**: EctC produced by *E*. *coli* after 0.2 mM IPTG induction; **7**: supernatant of lysed cells grown with nitrate and 0% NaCl; and **8**: supernatant of lysed cells grown with nitrate and 3% NaCl.

An ectoine synthesis gene cluster (*ectA*, *ectB*, and *ectC*) was identified in the ZD1 genome. The structure of the ectoine gene cluster in strain ZD1 was identical to those of some Firmicutes and Proteobacteria species (*Halomonas* sp. QHL1 [36], *Marinococcus halophilus* [37], and *Bacillus alcalophilus* DTY1 [11]. Expression of proteins (EctA, EctB, and EctC) in strain ZD1 grown under saline conditions was confirmed using SDS-PAGE analysis. As shown in Fig 6, the enzymes produced from the recombinant *E*. *coli* BL21 were used as positive controls; a band for EctA, 19 kilo-dalton (kDa) (lane 4), a band for EctB, 45 kDa (lane 5), and a band for EctC, 15 kDa (lane 6). Comparing to the positive control (lanes 4, 5, and 6) and 0% NaCl samples (lanes 2 and 7), ammonium sample with 3% NaCl showed EctB and EctA bands clearly (lane 3) and a clear band for EctB was shown in nitrate sample with 3% NaCl (lane 8). However, the faint band for EctC was only observed in ammonia sample with 3% NaCl (lane 3).

### Nitrogen metabolism

**Fig 7:**
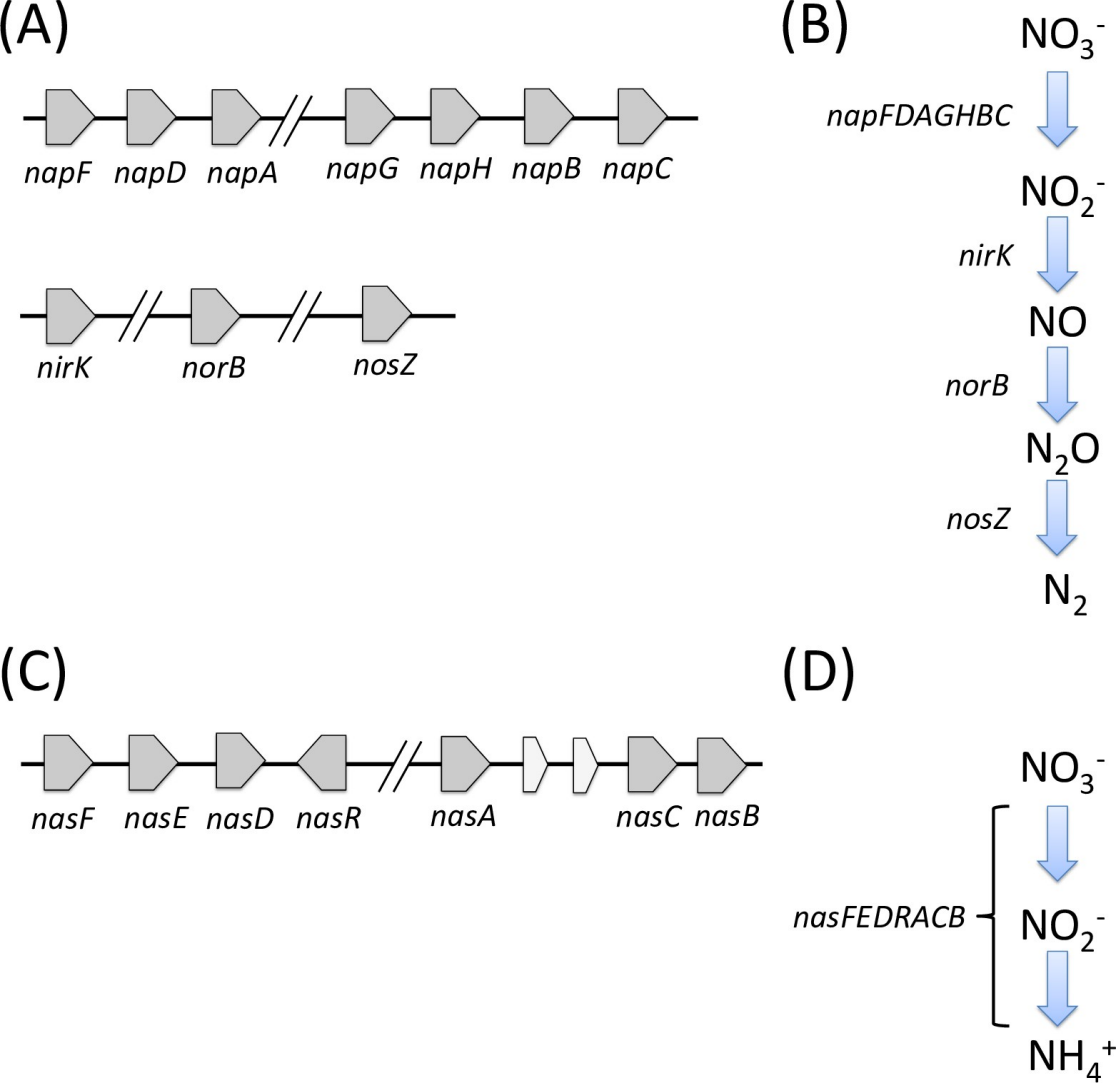
Genes and pathways of denitrification and assimilatory nitrate reduction. (A) Genes and gene clusters that play roles in denitrification. (B) A pathway to convert NO_3_^-^ to N_2_ adapted from previously studies [38–40]. (C) A gene cluster for assimilatory nitrate reduction. (D) A pathway for converting nitrate to ammonia.

Strain ZD1 has been shown to perform complete denitrification, i.e., converting nitrate to nitrogen gas and its ability is supported by a complete set of denitrifying genes identified in the ZD1 genome (Fig 7A). Fig 7B shows genes and gene clusters associated with denitrification process in ZD1. They are i) a gene cluster, *napFDAGHBC*, encoding a periplasmic nitrate reductase responsible for conversion of nitrate to nitrite; ii) a *nirK* gene encoding a copper-containing nitrite reductase which can convert nitrite to nitric oxide; iii) a *norB* gene encoding nitric oxide reductase subunit B that is involved in conversion of nitric oxide to nitrous oxide; and iv) a *nosZ* gene encoding nitrous-oxide reductase that is involved in conversion of nitrous oxide to nitrogen gas.

The structure of the periplasmic nitrate reductase nap gene cluster is very similar to those in *E*. *coli* [41], *Haemophilus influenza* [38], *Rhodoacter sphaeroides* [42], and *Magnetospirillum gryphiswaldense* [40]. NapF and NapD involve in post-translation of naturalization of NapA, while NapG, NapH, NapB and NapC assist electron transport to NapA (as demonstrated by Sparacino-Watkins et al. [43]). Two nitrite reductase genes (*nirK* and *nirS*) are functional biomarkers that are commonly used to investigate denitrifying bacteria [44]. In strain ZD1, only *nirK* was identified. The lack of a *norC* gene (which is usually found near or next to the *norB* gene) suggests that ZD1 may belong to single-component type of *norB* gene (quinol-oxidizing single-subunit class; qNorB) which is similar to that of *Ralstonia eutropha* H16 [45]. The phylogenetic tree of *qnorB* and *cnorB* (encoding cytochrome bc-type complex, cNorB) (S3 Fig) indicates that the ZD1 *norB* gene may be a single-component qNorB type.

An assimilatory nitrate reductase gene cluster (*nasFEDRACB*) was also identified in the ZD1 genome (Fig 7C and 7D). The cluster encodes enzymes involved in transport of nitrate or nitrite into the cytoplasm, followed by the conversion of nitrate to ammonia using *nasA*-encoded NADH-dependent nitrate and nitrite reductases [46]. This assimilatory nitrate reductase gene cluster is similar to that in *Klebsiella oxytoca* M5a1 (an assimilatory nitrate reductase gene cluster of *K*. *oxytoca* M5a1 is *nasRFEDCBA*) [46]. In *Klebsiella*, *nasFED* genes encode a typical ABC transporter for both nitrate and nitrite [47]. Thus, the presence of *nasFED* genes suggests that strain ZD1 might be able to uptake nitrate through cellular membranes. Differences in the structures of the assimilatory nitrate reductase gene clusters of ZD1 and *K*. *oxytoca* were observed. For example, the *nasR* gene, encoding an anti-terminator, is located in the downstream of the *nasFED* gene cluster, not the upstream. Additionally, *nasACB* genes are five genes away from the *nasRFED* gene cluster.

A two-gene cluster, including *nirB* and *nirD*, encoding a NADH-dependent nitrite reductase for converting nitrite to ammonia, was found in the ZD1 genome. This gene cluster structure is similar to those found in *E*. *coli* [48] and *Staphylococcus carnosus* [49]. *Mycobacterium tuberculosis* also possesses these two genes [50]. This nitrite reductase was reported to be mainly functional under anaerobic conditions [48, 49]; however, it was also found to be functional during aerobic growth in *M*. *tuberculosis* when nitrite was served as the sole nitrogen source [48, 49, 51]. Since ZD1 is a facultative anaerobe, we postulate that the nitrite reductase encoded by the *nirBD* gene cluster also functions under anaerobic conditions.

### Analysis of CRISPR-Cas system in strain ZD1

A CRISPR-Cas system, containing nine CRISPR genes along with a CRISPR array consisting of 117 spacers, was found in the ZD1 genome. The nine CRISPR genes included two universal CRISPR genes (*cas1* and *cas2*), five subtype-I-E genes (*cas6e*, *cas5*, *cas7*, *cse2gr11*, and *cas8e*), one type-I gene (*cas3HD*), and a WYL gene (*pfam13280*). The presence of the CRISPR-Cas system and the vast amount of spacers indicate that the ZD1 strain may have been a host for 117 different phages and thus acquired or developed defense mechanisms against them.

**Fig 8:**
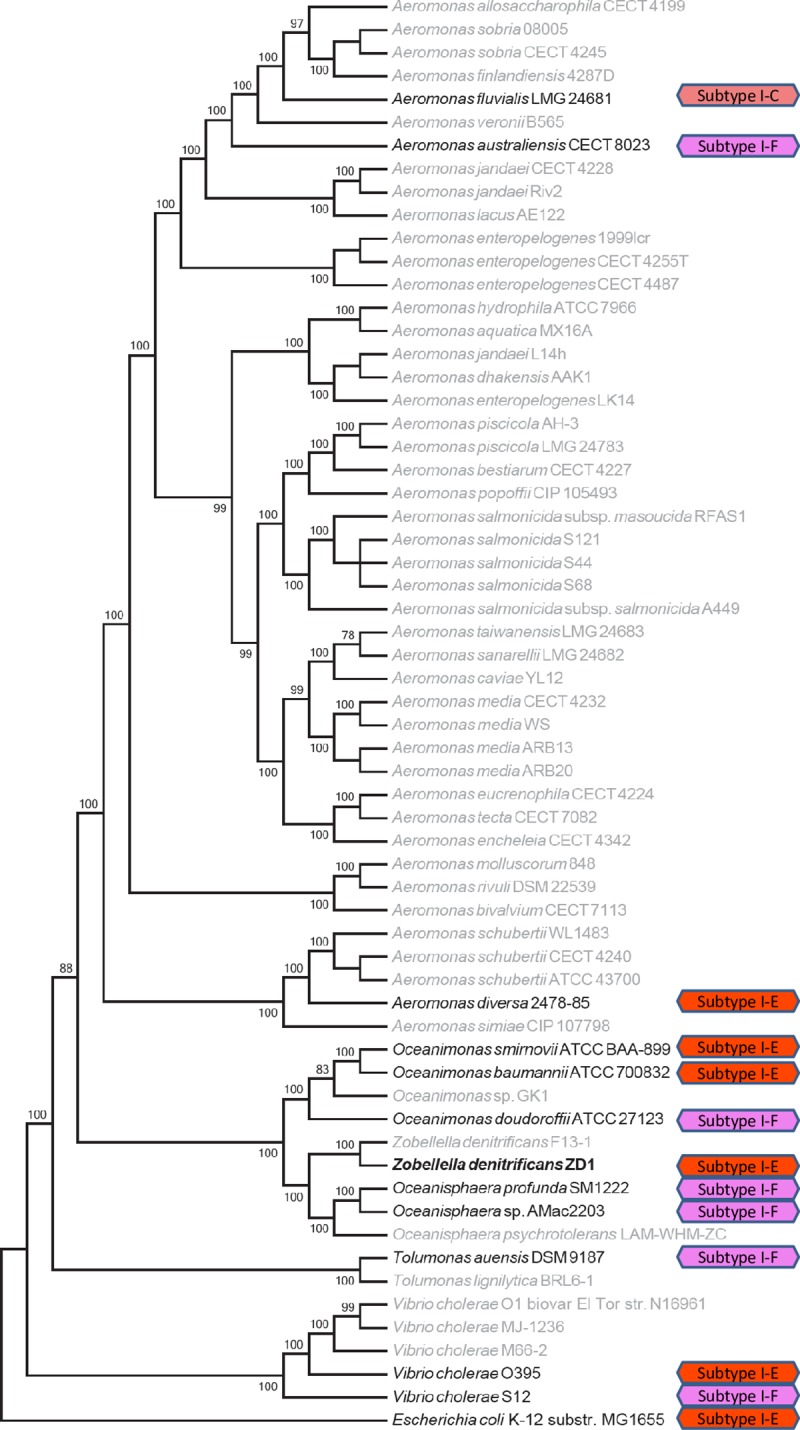
Identification of clustered regularly interspaced short palindromic repeat (CRISPR)-Cas systems from species closely related to *Zobellella denitrificans* ZD1 strain. Hexagonal boxes indicate identified CRISPR-Cas systems in these species. Different colors along with the text annotation represent different CRISPR systems.

As the genetic structure of this ZD1 CRISPR-Cas system is similar to that of *E*. *coli*, we searched some closely-related genomes, including the *Aeromonadaceae* family and *Vibrio cholerae*, in order to determine the distribution of this CRISPR-Cas system. Among 62 species, only 13 harbored the CRISPR-Cas system, including two *Aeromonas*, three *Oceanimonas*, two *Oceanisphaera*, one *Tolumonas*, two *Vibrio cholerae* species, *E*. *coli*, and *Z*. *denitrificans* strain ZD1. Fig 8 shows the distribution of the CRISIR-Cas system on the phylogenetic tree of the 62 species shown in Fig 1B. The results suggested that the CRISPR-Cas system may have been undergone extensive horizontal transfer events in the evolutionary history of the species. For example, the three *Oceanimonas* species do not share the same CRISPR system, in which two are subtype-I-E and one is subtype-I-F. The two closely related *Vibrio cholerae* species also harbor different CRISPR systems; not to mention that the three *Aeromonas* species contain three different CRISPR systems. Interestingly, we also observed that another *Z*. *denitrificans* strain F13-1 (referred as strain F13-1 hereafter), did not contain the CRISPR system, suggesting either that ZD1 horizontally acquired the CRISPR-Cas system from other species or that F13-1 may have lost this system.

**Fig 9:**
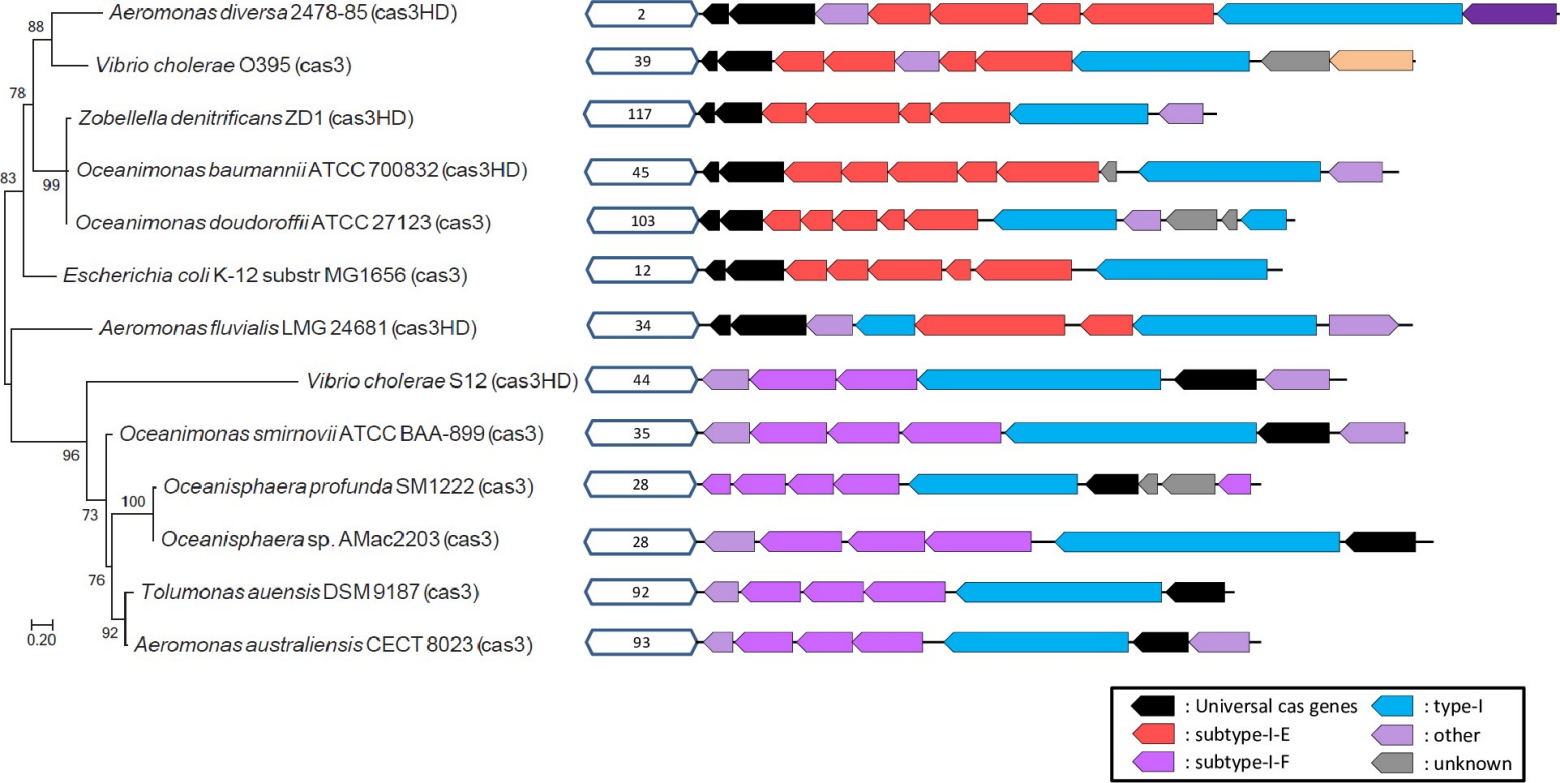
Phylogenetic trees built from the *cas3/cas3HD* genes of the clustered regularly interspaced short palindromic repeat (CRISPR)-Cas systems. Different colors indicate different types of CRISPR genes, and white hexagons in front of the CRISPR-Cas genes contain the numbers of repeats (i.e., the number of spacers) identified from the CRISPR system.

An evolutionary tree for the *cas3* gene was built, as this gene is shared by all probed species containing the CRISPR system. As shown in Fig 9, the CRISPR-Cas systems of strain ZD1 and *E*. *coli* are not very closely related despite their highly similar gene order. Instead, the *cas3* gene of strain ZD1 is most closely related to those of two *Oceanimonas* species, suggesting that *Z*. *denitrificans* may have inherited the CRISPR-Cas system from the common ancestors, and that strain F13-1 may have recently lost the CRISPR-Cas system. The cross-comparison between the *cas3* tree (Fig 9) and the CRISPR-marked phylogenetic tree (Fig 8) further suggests that CRISPR-Cas systems have been extensively exchanged among different oceanic species.

**Fig 10:**
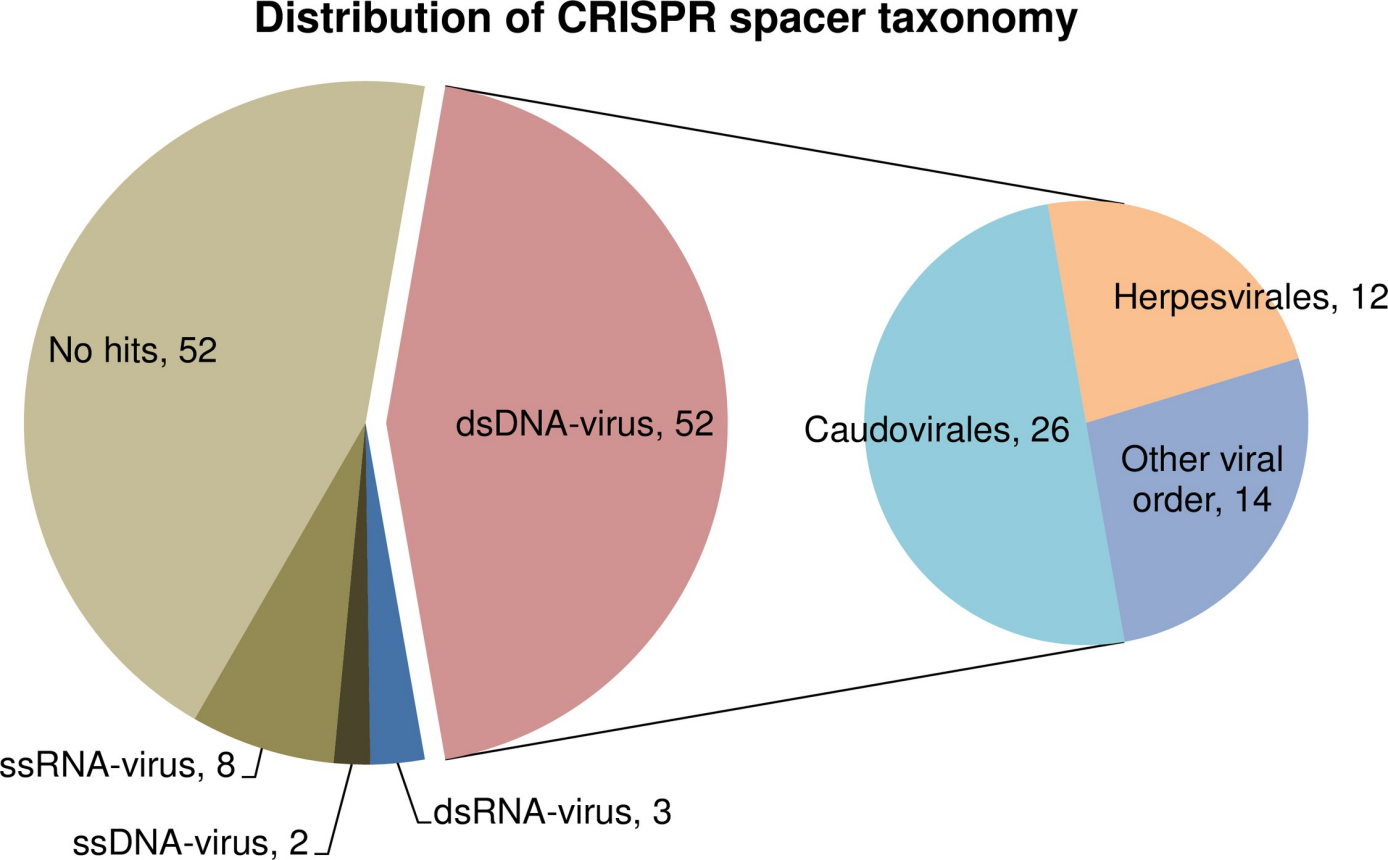
Taxonomic distribution of clustered regularly interspaced short palindromic repeat (CRISPR) spacers.

The taxonomic distribution of the ZD1 CRISPR spacers was also explored in order to determine phages that may commonly infect this species. As shown in Fig 10, 52 out of 117 spacers (i.e., 44%) belonged to double-stranded (ds) DNA viruses, and almost half of the dsDNA viruses were Caudovirales. The other 12% belongs to ssRNA-virus, ssDNA-virus, and dsRNA-virus. However, another 44% of spacers could not be identified using available viral sequences database. The diversification of viral spacers in the ZD1 CRISPR spacers suggested that strain ZD1 may have been the host target of a variety of phages. CRISP-Cas is an immune system allows a bacterium to fight against phage infection. Bacteria incorporate small fragments of phage DNA/RNA into their genome as spacers which were derived from the phages that the host bacteria previously infected. In the event of subsequent infection, these spacers work together with the Cas protein to recognize and digest the phage DNA/RNA. In this study, the high number of spacers found in the ZD1 genome would allow the strain to fight against infection of phages that they previously infected and such a strong phage immune system in the strain might explain the fail attempts in our previous efforts in isolating environmental phages to infect stain ZD1.

## Discussion

We have successfully analyzed the genome of strain ZD1 and acquired a better understanding of its genetic materials that are responsible for complete denitrification and for converting crude glycerol to PHB using a complete glycerol-to-PHB pathway. The identification and characterization of a four-gene-cluster for PHB synthesis that was found in marine-specific Gammaproteobacteria species and elevated expression of PHB synthesis genes might explain why strain ZD1 can effectively synthesize PHB in saline environments. With the availability and low price of crude glycerol in recent years [52], understanding the complete pathway for oxidizing glycerol into PHB by strain ZD1 open a new door for producing PHB economically.

Sterilization of culture media to prevent microbial contamination during industrial production of desired bioproducts, including PHB, is a costly process. To overcome this challenge, recent studies explored the feasibility of using halophilic archaeal strains to produce PHB using non-sterile media that contain high-salt contents [53, 54]. The proteins expressed from the ectoine synthesis gene cluster (*ectA*, *ectB*, and *ectC*) in the ZD1 offer a growth advantage of ZD1 over other non-salt-tolerant strains under high-saline culture conditions. Our previous works showed that in 3% salt condition, the ZD1 can accumulate PHB up to 3.61 g/L by using glycerol and nitrate as carbon and nitrogen source respectively (C/N ratio: 21.5) [12] implying the feasibility of non-sterile, large-scale production of PHB by ZD1.

Given the well-known complete denitrification ability of *Z*. *denitrificans*, it is not surprising to identify genes/gene clusters (such as *napFDAGHBC*, *nirK*, *norB*, and *nosZ*) responsible for a complete denitrification pathway in the ZD1 genome. Additionally, genes for assimilatory nitrate reduction were identified, suggesting that ZD1 may be able to convert nitrate to nitrogen gas or to ammonia. However, it was unclear under what conditions that strain ZD1 utilizes nitrate for denitrification or for assimilatory nitrate reduction. Since certain organisms (for example, *Wolinella succinogenes*) are able to reduce nitrate through an alternative pathway [55], ZD1 may possess the ability to switch between or cross-utilize both pathways to achieve the full capacity of taking up nitrate from the surrounding environment.

No detection of *amoA* genes (a biomarker used to identify microorganisms capable of oxidizing ammonia to nitrite [56]) in the ZD1 genome and an observation of well growth of ZD1 with glycerol and ammonia suggested that ammonia was utilized as a nitrogen source. A cytidine triphosphate (CTP) synthase was discovered in the ZD1 genome, which may play a role in catalyzing the formation of CTP from uridine-5'-triphosphate (UTP) using either ammonia or l-glutamine as the nitrogen source [57]. This discovery implies that ZD1 may utilize ammonia for the biosynthesis of nucleic acid [58]. Kilstrup et al. also mentioned that activated intermediates produced in the conversion process of UTP to CTP reacted with an ammonia molecule in order to synthesize CTP [59]. Future experimental or analytical works are needed to identify the actual genetic mechanisms for ZD1 to utilize ammonia for growth and potential PHB synthesis purpose.

In this study, we observed that the expression of the PHB synthesis related genes (i.e., *phaABC* and the *PFP*) were significantly increased in nitrate-grown cultures compared to those grown with ammonium. These observations might be explained by that fact that strain ZD1 is able to transport nitrate through membrane for nitrogen source (via an assimilatory nitrate reductase gene cluster (*nasFEDRACB*) as shown in Fig 7) and thus has a better fitness using nitrate as nitrogen source for cell growth, which in turn results in efficient utilization and conversion of glycerol to PHB. Yet, future studies are needed to further investigate the regulation and interactions among nitrogen metabolism, glycerol utilization, PHB accumulation under different saline conditions.

The high number of spacers (117 total) in the CRISPR-Cas system in strain ZD1 suggested that strain ZD1 may have an immune system capable of against a variety of phages. The investigation of the CRISPR-Cas system and its distribution among members of the *Aeromonadaceae* family suggests that the CRISPR-Cas system may very frequently be exchanged or horizontally transferred among oceanic bacteria. Unlike the elaborate CRISPR-Cas system identified in strain ZD1, a surprising discovery is that there is no CRISPR-Cas system present in strain F13-1. Given the vast amount of spacers in the CRISPR-Cas system identified in the ZD1 genome, it is unlikely that ZD1 acquired the CRISPR-Cas system very recently. A plausible explanation may be that strain F13-1 dropped the CRISPR-Cas system and may become more vulnerable to phage attacks.

The phylogenetic tree built from *cas3* genes showed that the ZD1 CRISPR was more closely related to those of *Oceanimonas baumannii* ATCC 700832 and *Oceanimonas doudoroffii* ATCC 27123. The differences between the ZD1 CRISPR-Cas system and those of the two *Oceanimonas* species also suggest that the CRISPR-Cas system may have undergone fast gene content changes. The changes in CRISPR gene content may reflect the co-evolution of bacteria and the phages that attack them, in which phages need to escape the recognition of the CRISPR system while the bacteria need to catch more phage sequences through their CRISPR defense system [60]. Westra et al. [61] also suggested that CRISPR loci can evolve very rapidly in natural environments. More research in the CRISPR content change with the bacteria-virus co-evolution theme may lead to more interesting discoveries in the CRISPR research realm.

## Conclusions

We have identified a complete PHB synthesis pathway from glycerol to PHB in the strain ZD1 genome. The novel PHB synthesis gene cluster with a phasin-family protein in the ZD1 genome provides new insights into how *Zobellella* species synthesize PHB. High PHB production and elevated expression of PHB synthesis genes in ZD1 grown with glycerol and nitrate suggested the important linkage of PHB synthesis genes and nitrogen source. The ability of strain ZD1 to grow under high salt conditions has also experimentally validated through the production of proteins responsible for ectoine synthesis. Characterization of the complete denitrification pathway explained how strain ZD1 performs denitrification. The results of this study enhance our knowledge about high PHB production by marine organisms. Overall, our findings suggest that using strain ZD1 for non-sterile PHB production from inexpensive, non-sterile growth substrate can be a viable new approach to economic PHB production in the future.

## Supporting information

S1 Appendix List of abbreviations. (DOCX)

S2 Appendix Availability of data and materials. (DOCX)

S1 Fig 16S rDNA-based tree comparing *Zobellella denitrificans* and *Zobellella taiwanensis*, the first two members discovered in the *Zobellella* family. (DOCX)

S2 Fig Phasin gene functional domain analysis and comparison. (A) Conserved functional domain predicted from the phasin gene within the *phaC*, -*A*, and -*B* gene cluster and the *phpP* phasin gene. (B) Phylogenetic tree built on phasin-family genes.
(DOCX)

S3 Fig Phylogenetic tree based on *norB* genes. (DOCX)

S1 Table Primer sets used for RT-PCR and qPCR analysis of PHB synthesis genes and housekeeping gene (16S rRNA) in *Zobellella denitrificans* ZD1. (DOCX)

S2 Table Single copy marker genes used for building the concatenated genome tree as shown in Fig 1(B). See Materials and Methods for details.
(DOCX)
