# Supplementary material for: Analysis of Zobellella denitrificans ZD1 draft genome: Genes and gene clusters responsible for high polyhydroxybutyrate (PHB) production from glycerol under saline conditions and its CRISPR-Cas system
Source: PLoS One. 2019 Sep 12;14(9):e0222143. doi: 10.1371/journal.pone.0222143 (PMC6742469; doi:10.1371/journal.pone.0222143)
Supplement: S2 Appendix — (DOCX) [file pone.0222143.s002.docx]

**Supporting Information**

**for**

**Analysis of *Zobellella denitrificans* ZD1 draft genome: Genes and gene clusters responsible for high polyhydroxybutyrate (PHB) production from glycerol under saline conditions and its CRISPR-Cas system**

Yu-Wei Wu^1,2*^, Shih-Hung Yang^3^, Myung Hwangbo^3^, and Kung-Hui Chu^3*^

^1^Graduate Institute of Biomedical Informatics, College of Medical Science and Technology, Taipei Medical University, Taipei 106, Taiwan; ^2^Clinical Big Data Research Center, Taipei Medical University Hospital, Taipei 110, Taiwan; ^3^Zachry Department of Civil and Environmental Engineering, Texas A&M University, College Station, TX77843, USA

**S2 Appendix. Availability of data and materials**

The accession number for the *Z. denitrificans* ZD1 draft genome is NMUO00000000.
