# Supplementary material for: Analysis of Zobellella denitrificans ZD1 draft genome: Genes and gene clusters responsible for high polyhydroxybutyrate (PHB) production from glycerol under saline conditions and its CRISPR-Cas system
Source: PLoS One. 2019 Sep 12;14(9):e0222143. doi: 10.1371/journal.pone.0222143 (PMC6742469; doi:10.1371/journal.pone.0222143)
Supplement: S1 Table — (DOCX) [file pone.0222143.s006.docx]

**Supporting Information**

**for**

**Analysis of *Zobellella denitrificans* ZD1 draft genome: Genes and gene clusters responsible for high polyhydroxybutyrate (PHB) production from glycerol under saline conditions and its CRISPR-Cas system**

Yu-Wei Wu^1,2*^, Shih-Hung Yang^3^, Myung Hwangbo^3^, and Kung-Hui Chu^3*^

^1^Graduate Institute of Biomedical Informatics, College of Medical Science and Technology, Taipei Medical University, Taipei 106, Taiwan; ^2^Clinical Big Data Research Center, Taipei Medical University Hospital, Taipei 110, Taiwan; ^3^Zachry Department of Civil and Environmental Engineering, Texas A&M University, College Station, TX77843, USA

**S1 Table. Primer sets used for RT-PCR and qPCR analysis of PHB synthesis genes and housekeeping gene (16S rRNA) in *Zobellella denitrificans* ZD1.**

**S1 Table. Primer sets used for RT-PCR and qPCR analysis of PHB synthesis genes and housekeeping gene (16S rRNA) in *Zobellella denitrificans* ZD1.**

| Target Gene | Primers | Sequence | Product length (bp) | Tm | Reference |
| --- | --- | --- | --- | --- | --- |
| *phaA* | phaA-F | 5’-ACCATGTTCAGGGTGTAAGC-3’ | 126 | 54 | This study |
|  | phaA-R | 5’-GGACAAGGTCAACGAAGTGA-3’ |  |  |  |
| *phaB* | phaB-F | 5’-TGGTGGCCTGTTGTTCTTT-3’ | 95 | 54 | This study |
|  | phaB-R | 5’-CGGTGGAATAGGAACAGAAGTG-3’ |  |  |  |
| *phaC* | phaC-F | 5’-ATGGCGTTCACTTCCTTCTC-3’ | 146 | 54 | This study |
|  | phaC-R | 5’-CAGGGCCATACGGTCTTTATC-3’ |  |  |  |
| *pfp* | Pfp-F | 5’-CATGGTTTCCAGTTGCTTGG-3’ | 140 | 54 | This study |
|  | Pfp-R | 5’-ACTTCACCGATCTGACCAAC-3’ |  |  |  |
| 16S rRNA | 16S-F | 5’-ATGGCTGTCGTCAGCT-3’ | 352 | 50 | [[1](#_ENREF_1)] |
|  | 16S-R | 5’-ACGGGCGGTGTGTAC-3’ |  |  |  |

Reference

1. Ferris MJ, Muyzer G, Ward DM. Denaturing gradient gel electrophoresis profiles of 16S rRNA-defined populations inhabiting a hot spring microbial mat community. Appl Environ Microbiol. 1996;62:340-346.
