# Supplementary material for: Analysis of Zobellella denitrificans ZD1 draft genome: Genes and gene clusters responsible for high polyhydroxybutyrate (PHB) production from glycerol under saline conditions and its CRISPR-Cas system
Source: PLoS One. 2019 Sep 12;14(9):e0222143. doi: 10.1371/journal.pone.0222143 (PMC6742469; doi:10.1371/journal.pone.0222143)
Supplement: S2 Table — See Materials and Methods for details. (DOCX) [file pone.0222143.s007.docx]

**Supporting Information**

**for**

**Analysis of *Zobellella denitrificans* ZD1 draft genome: Genes and gene clusters responsible for high polyhydroxybutyrate (PHB) production from glycerol under saline conditions and its CRISPR-Cas system**

Yu-Wei Wu^1,2*^, Shih-Hung Yang^3^, Myung Hwangbo^3^, and Kung-Hui Chu^3*^

^1^Graduate Institute of Biomedical Informatics, College of Medical Science and Technology, Taipei Medical University, Taipei 106, Taiwan; ^2^Clinical Big Data Research Center, Taipei Medical University Hospital, Taipei 110, Taiwan; ^3^Zachry Department of Civil and Environmental Engineering, Texas A&M University, College Station, TX77843, USA

**S2 Table. Single copy marker genes used for building the concatenated genome tree as shown in Fig 1(B).**

**S2 Table. Single copy marker genes used for building the concatenated genome tree as shown in Fig 1(B).** See Materials and Methods for details.

| Pfam accession | Pfam summary |
| --- | --- |
| PF04077.11 | DsrH, DsrH |
| PF06804.10 | Lipoprotein_18, NlpB/DapX |
| PF05494.11 | MlaC, MlaC |
| PF13167.5 | GTP-bdg_N, GTP-binding |
| PF01250.16 | Ribosomal_S6, Ribosomal |
| PF14819.5 | QueF_N, Nitrile |
| PF00573.21 | Ribosomal_L4, Ribosomal |
| PF01245.19 | Ribosomal_L19, Ribosomal |
| PF00227.25 | Proteasome, Proteasome |
| PF06667.11 | PspB, Phage |
| PF00764.18 | Arginosuc_synth, Arginosuccinate |
| PF04608.12 | PgpA, Phosphatidylglycerophosphatase |
| PF13525.5 | YfiO, Outer |
| PF07208.10 | DUF1414, Protein |
| PF04445.12 | SAM_MT, Putative |
| PF01678.18 | DAP_epimerase, Diaminopimelate |
| PF17136.3 | ribosomal_L24, Ribosomal |
| PF02609.15 | Exonuc_VII_S, Exonuclease |
| PF04217.12 | DUF412, Protein |
| PF00218.20 | IGPS, Indole-3-glycerol |
| PF01491.15 | Frataxin_Cyay, Frataxin-like |
| PF01295.17 | Adenylate_cycl, Adenylate |
| PF01967.20 | MoaC, MoaC |
| PF04127.14 | DFP, DNA |
| PF02575.15 | YbaB_DNA_bd, YbaB/EbfC |
| PF00344.19 | SecY, SecY |
| PF00476.19 | DNA_pol_A, DNA |
| PF02445.15 | NadA, Quinolinate |
| PF03947.17 | Ribosomal_L2_C, Ribosomal |
| PF17432.1 | DUF3458_C, Domain |
| PF01795.18 | Methyltransf_5, MraW |
| PF00136.20 | DNA_pol_B, DNA |
| PF00253.20 | Ribosomal_S14, Ribosomal |
| PF02272.18 | DHHA1, DHHA1 |
| PF01985.20 | CRS1_YhbY, CRS1 |
| PF12344.7 | UvrB, Ultra-violet |
| PF04186.12 | FxsA, FxsA |
| PF01761.19 | DHQ_synthase, 3-dehydroquinate |
| PF14622.5 | Ribonucleas_3_3, Ribonuclease-III-like |
| PF02631.15 | RecX, RecX |
| PF00164.24 | Ribosom_S12_S23, Ribosomal |
| PF03872.12 | RseA_N, Anti |
| PF00831.22 | Ribosomal_L29, Ribosomal |
| PF00471.19 | Ribosomal_L33, Ribosomal |
| PF00406.21 | ADK, Adenylate |
| PF02113.14 | Peptidase_S13, D-Ala-D-Ala |
| PF03099.18 | BPL_LplA_LipB, Biotin/lipoate |
| PF06005.11 | ZapB, Cell |
| PF00189.19 | Ribosomal_S3_C, Ribosomal |
| PF01430.18 | HSP33, Hsp33 |
| PF13369.5 | Transglut_core2, Transglutaminase-like |
| PF02664.14 | LuxS, S-Ribosylhomocysteinase |
| PF02091.14 | tRNA-synt_2e, Glycyl-tRNA |
| PF08298.10 | AAA_PrkA, PrkA |
| PF04378.12 | RsmJ, Ribosomal |
| PF02576.16 | DUF150, RimP |
| PF02092.16 | tRNA_synt_2f, Glycyl-tRNA |
| PF06426.13 | SATase_N, Serine |
| PF13464.5 | DUF4115, Domain |
| PF03740.12 | PdxJ, Pyridoxal |
| PF02699.14 | YajC, Preprotein |
| PF06794.11 | UPF0270, Uncharacterised |
| PF02578.14 | Cu-oxidase_4, Multi-copper |
| PF02955.15 | GSH-S_ATP, Prokaryotic |
| PF08459.10 | UvrC_HhH_N, UvrC |
| PF02976.14 | MutH, DNA |
| PF14561.5 | TPR_20, Tetratricopeptide |
| PF02540.16 | NAD_synthase, NAD |
| PF01668.17 | SmpB, SmpB |
| PF13116.5 | DUF3971, Protein |
| PF06325.12 | PrmA, Ribosomal |
| PF02569.14 | Pantoate_ligase, Pantoate-beta-alanine |
| PF08496.9 | Peptidase_S49_N, Peptidase |
| PF07991.11 | IlvN, Acetohydroxy |
| PF00318.19 | Ribosomal_S2, Ribosomal |
| PF03486.13 | HI0933_like, HI0933-like |
| PF03948.13 | Ribosomal_L9_C, Ribosomal |
| PF04241.14 | DUF423, Protein |
| PF06835.12 | LptC, Lipopolysaccharide-assembly, |
| PF00238.18 | Ribosomal_L14, Ribosomal |
| PF01746.20 | tRNA_m1G_MT, tRNA |
| PF04361.12 | DUF494, Protein |
| PF01728.18 | FtsJ, FtsJ-like |
| PF01948.17 | PyrI, Aspartate |
| PF08335.10 | GlnD_UR_UTase, GlnD |
| PF09382.9 | RQC, RQC |
| PF08529.10 | NusA_N, NusA |
| PF01725.15 | Ham1p_like, Ham1 |
| PF01018.21 | GTP1_OBG, GTP1/OBG |
| PF08411.9 | Exonuc_X-T_C, Exonuclease |
| PF12843.6 | QSregVF_b, Putative |
| PF04222.11 | DUF416, Protein |
| PF04364.12 | DNA_pol3_chi, DNA |
| PF16331.4 | TolA_bind_tri, TolA |
| PF02556.13 | SecB, Preprotein |
| PF01142.17 | TruD, tRNA |
| PF00687.20 | Ribosomal_L1, Ribosomal |
| PF03796.14 | DnaB_C, DnaB-like |
| PF02660.14 | G3P_acyltransf, Glycerol-3-phosphate |
| PF11808.7 | DUF3329, Domain |
| PF00750.18 | tRNA-synt_1d, tRNA |
| PF02527.14 | GidB, rRNA |
| PF04452.13 | Methyltrans_RNA, RNA |
| PF04380.12 | BMFP, Membrane |
| PF03776.13 | MinE, Septum |
| PF00162.18 | PGK, Phosphoglycerate |
| PF01765.18 | RRF, Ribosome |
| PF03364.19 | Polyketide_cyc, Polyketide |
| PF07743.12 | HSCB_C, HSCB |
| PF01964.17 | ThiC_Rad_SAM, Radical |
| PF04551.13 | GcpE, GcpE |
| PF00297.21 | Ribosomal_L3, Ribosomal |
| PF01502.17 | PRA-CH, Phosphoribosyl-AMP |
| PF14849.5 | YidC_periplas, YidC |
| PF10369.8 | ALS_ss_C, Small |
| PF00925.19 | GTP_cyclohydro2, GTP |
| PF00694.18 | Aconitase_C, Aconitase |
| PF14681.5 | UPRTase, Uracil |
| PF03883.13 | H2O2_YaaD, Peroxide |
| PF03923.12 | Lipoprotein_16, Uncharacterized |
| PF01196.18 | Ribosomal_L17, Ribosomal |
| PF01416.19 | PseudoU_synth_1, tRNA |
| PF04288.12 | MukE, MukE-like |
| PF08349.10 | DUF1722, Protein |
| PF13624.5 | SurA_N_3, SurA |
| PF03331.12 | LpxC, UDP-3-O-acyl |
| PF01782.17 | RimM, RimM |
| PF01702.17 | TGT, Queuine |
| PF01980.15 | UPF0066, Uncharacterised |
| PF04219.11 | DUF413, Protein |
| PF04354.12 | ZipA_C, ZipA, |
| PF02620.16 | DUF177, Uncharacterized |
| PF02639.13 | DUF188, Uncharacterized |
| PF04102.11 | SlyX, SlyX |
| PF05496.11 | RuvB_N, Holliday |
| PF00988.21 | CPSase_sm_chain, Carbamoyl-phosphate |
| PF07126.11 | ZapC, Cell-division |
| PF03884.13 | YacG, DNA |
| PF04357.12 | TamB, TamB, |
| PF07219.12 | HemY_N, HemY |
| PF03255.13 | ACCA, Acetyl |
| PF02739.15 | 5_3_exonuc_N, 5'-3' |
| PF07733.11 | DNA_pol3_alpha, Bacterial |
| PF03799.14 | FtsQ, Cell |
| PF11892.7 | DUF3412, Domain |
| PF01165.19 | Ribosomal_S21, Ribosomal |
| PF07348.11 | Syd, Syd |
| PF01135.18 | PCMT, Protein-L-isoaspartate(D-aspartate) |
| PF03461.14 | TRCF, TRCF |
| PF00366.19 | Ribosomal_S17, Ribosomal |
| PF13522.5 | GATase_6, Glutamine |
| PF01784.17 | NIF3, NIF3 |
| PF05164.12 | ZapA, Cell |
| PF07840.11 | FadR_C, FadR |
| PF03453.16 | MoeA_N, MoeA |
| PF02153.16 | PDH, Prephenate |
| PF03550.13 | LolB, Outer |
| PF00213.17 | OSCP, ATP |
| PF06865.10 | DUF1255, Protein |
| PF03925.12 | SeqA, SeqA |
| PF01255.18 | Prenyltransf, Putative |
| PF00154.20 | RecA, recA |
| PF00825.17 | Ribonuclease_P, Ribonuclease |
| PF05690.13 | ThiG, Thiazole |
| PF00347.22 | Ribosomal_L6, Ribosomal |
| PF01351.17 | RNase_HII, Ribonuclease |
| PF02590.16 | SPOUT_MTase, Predicted |
| PF05166.12 | YcgL, YcgL |
| PF02391.16 | MoaE, MoaE |
| PF01084.19 | Ribosomal_S18, Ribosomal |
| PF02390.16 | Methyltransf_4, Putative |
| PF04246.11 | RseC_MucC, Positive |
| PF00163.18 | Ribosomal_S4, Ribosomal |
| PF04461.12 | DUF520, Protein |
| PF01434.17 | Peptidase_M41, Peptidase |
| PF03937.15 | Sdh5, Flavinator |
| PF02866.17 | Ldh_1_C, lactate/malate |
| PF03668.14 | ATP_bind_2, P-loop |
| PF02580.15 | Tyr_Deacylase, D-Tyr-tRNA(Tyr) |
| PF03888.13 | MucB_RseB, MucB/RseB |
| PF01106.16 | NifU, NifU-like |
| PF04359.13 | DUF493, Protein |
| PF01790.17 | LGT, Prolipoprotein |
| PF13023.5 | HD_3, HD |
| PF12137.7 | RapA_C, RNA |
| PF00276.19 | Ribosomal_L23, Ribosomal |
| PF00237.18 | Ribosomal_L22, Ribosomal |
| PF13393.5 | tRNA-synt_His, Histidyl-tRNA |
| PF03968.13 | OstA, OstA-like |
| PF01029.17 | NusB, NusB |
| PF00203.20 | Ribosomal_S19, Ribosomal |
| PF09976.8 | TPR_21, Tetratricopeptide |
| PF00707.21 | IF3_C, Translation |
| PF01977.15 | UbiD, 3-octaprenyl-4-hydroxybenzoate |
| PF00828.18 | Ribosomal_L27A, Ribosomal |
| PF07479.13 | NAD_Gly3P_dh_C, NAD-dependent |
| PF02617.16 | ClpS, ATP-dependent |
| PF01808.17 | AICARFT_IMPCHas, AICARFT/IMPCHase |
| PF00542.18 | Ribosomal_L12, Ribosomal |
| PF00830.18 | Ribosomal_L28, Ribosomal |
| PF14450.5 | FtsA, Cell |
| PF01632.18 | Ribosomal_L35p, Ribosomal |
| PF02325.16 | YGGT, YGGT |
| PF06574.11 | FAD_syn, FAD |
| PF02547.14 | Queuosine_synth, Queuosine |
| PF00453.17 | Ribosomal_L20, Ribosomal |
| PF01016.18 | Ribosomal_L27, Ribosomal |
| PF02670.15 | DXP_reductoisom, 1-deoxy-D-xylulose |
| PF04085.13 | MreC, rod |
| PF02033.17 | RBFA, Ribosome-binding |
| PF01121.19 | CoaE, Dephospho-CoA |
| PF00466.19 | Ribosomal_L10, Ribosomal |
| PF00584.19 | SecE, SecE/Sec61-gamma |
| PF03755.12 | YicC_N, YicC-like |
| PF01208.16 | URO-D, Uroporphyrinogen |
| PF01330.20 | RuvA_N, RuvA |
| PF01195.18 | Pept_tRNA_hydro, Peptidyl-tRNA |
| PF01379.19 | Porphobil_deam, Porphobilinogen |
| PF03652.14 | RuvX, Holliday |
| PF01205.18 | UPF0029, Uncharacterized |
| PF11398.7 | DUF2813, Protein |
| PF01025.18 | GrpE, GrpE |
| PF00490.20 | ALAD, Delta-aminolevulinic |
| PF00410.18 | Ribosomal_S8, Ribosomal |
| PF04353.12 | Rsd_AlgQ, Regulator |
| PF03946.13 | Ribosomal_L11_N, Ribosomal |
| PF00625.20 | Guanylate_kin, Guanylate |
| PF03938.13 | OmpH, Outer |
| PF07295.10 | DUF1451, Zinc-ribbon |
| PF02684.14 | LpxB, Lipid-A-disaccharide |
| PF04093.11 | MreD, rod |
| PF01634.17 | HisG, ATP |
| PF00475.17 | IGPD, Imidazoleglycerol-phosphate |
| PF00889.18 | EF_TS, Elongation |
| PF04352.12 | ProQ, ProQ/FINO |
| PF02601.14 | Exonuc_VII_L, Exonuclease |
| PF02367.16 | TsaE, Threonylcarbamoyl |
| PF03840.13 | SecG, Preprotein |
| PF04999.12 | FtsL, Cell |
| PF01192.21 | RNA_pol_Rpb6, RNA |
| PF08331.9 | DUF1730, Domain |
| PF02021.16 | UPF0102, Uncharacterised |
| PF04340.11 | DUF484, Protein |
| PF02130.16 | UPF0054, Uncharacterized |
| PF01071.18 | GARS_A, Phosphoribosylglycinamide |
| PF01509.17 | TruB_N, TruB |
| PF06062.10 | UPF0231, Uncharacterised |
| PF04453.13 | OstA_C, Organic |
| PF02568.13 | ThiI, Thiamine |
| PF03588.13 | Leu_Phe_trans, Leucyl/phenylalanyl-tRNA |
| PF01726.15 | LexA_DNA_bind, LexA |
| PF04287.11 | DUF446, tRNA |
| PF01227.21 | GTP_cyclohydroI, GTP |
| PF06305.10 | LapA_dom, Lipopolysaccharide |
| PF01119.18 | DNA_mis_repair, DNA |
| PF02873.15 | MurB_C, UDP-N-acetylenolpyruvoylglucosamine |
| PF02224.17 | Cytidylate_kin, Cytidylate |
| PF07264.10 | EI24, Etoposide-induced |
| PF04348.12 | LppC, LppC |
| PF04345.12 | Chor_lyase, Chorismate |
| PF03023.13 | MVIN, MviN-like |
| PF00334.18 | NDK, Nucleoside |
| PF01715.16 | IPPT, IPP |
| PF00312.21 | Ribosomal_S15, Ribosomal |
| PF00958.21 | GMP_synt_C, GMP |
| PF04375.13 | HemX, HemX, |
| PF08482.9 | HrpB_C, ATP-dependent |
| PF12631.6 | MnmE_helical, MnmE |
| PF00673.20 | Ribosomal_L5_C, ribosomal |
| PF09371.9 | Tex_N, Tex-like |
| PF00488.20 | MutS_V, MutS |
| PF01176.18 | eIF-1a, Translation |
| PF09984.8 | DUF2222, Uncharacterised |
| PF01259.17 | SAICAR_synt, SAICAR |
| PF04362.13 | Iron_traffic, Bacterial |
| PF02674.15 | Colicin_V, Colicin |
| PF05127.13 | Helicase_RecD, Helicase |
| PF06026.13 | Rib_5-P_isom_A, Ribose |
| PF06071.12 | YchF-GTPase_C, Protein |
| PF02696.13 | UPF0061, Uncharacterized |
| PF01134.21 | GIDA, Glucose |
| PF02773.15 | S-AdoMet_synt_C, S-adenosylmethionine |
| PF01649.17 | Ribosomal_S20p, Ribosomal |
| PF02410.14 | RsfS, Ribosomal |
| PF02592.14 | Vut_1, Putative |
| PF04386.12 | SspB, Stringent |
| PF11898.7 | DUF3418, Domain |
